# *BRCA1* and *BRCA2* mutational profile and prevalence in hereditary breast and ovarian cancer (HBOC) probands from Southern Brazil: Are international testing criteria appropriate for this specific population?

**DOI:** 10.1371/journal.pone.0187630

**Published:** 2017-11-21

**Authors:** Bárbara Alemar, Cleandra Gregório, Josef Herzog, Camila Matzenbacher Bittar, Cristina Brinckmann Oliveira Netto, Osvaldo Artigalas, Ida Vanessa D. Schwartz, Jordy Coffa, Suzi Alves Camey, Jeffrey Weitzel, Patricia Ashton-Prolla

**Affiliations:** 1 Programa de Pós-graduação em Genética e Biologia Molecular, Universidade Federal do Rio Grande do Sul, Porto Alegre, Rio Grande do Sul, Brazil; 2 Laboratório de Medicina Genômica, Hospital de Clínicas de Porto Alegre, Porto Alegre, Rio Grande do Sul, Brazil; 3 Department of Population Sciences, Division of Clinical Cancer Genomics, City of Hope, Duarte, California, United States of America; 4 Serviço de Genética Médica, Hospital de Clínicas de Porto Alegre, Porto Alegre, Rio Grande do Sul, Brazil; 5 Hospital Moinhos de Vento, Porto Alegre, Rio Grande do Sul, Brazil; 6 Departamento de Genética, Universidade Federal do Rio Grande do Sul, Porto Alegre, Rio Grande do Sul, Brazil; 7 MRC-Holland, Amsterdam, the Netherlands; 8 Programa de Pós-graduação em Epidemiologia, Departamento de Estatística, Universidade Federal do Rio Grande do Sul, Porto Alegre, Rio Grande do Sul, Brazil; Ohio State University Wexner Medical Center, UNITED STATES

## Abstract

**Background:**

Germline pathogenic variants in *BRCA1* and *BRCA2* (*BRCA*) are the main cause of Hereditary Breast and Ovarian Cancer syndrome (HBOC).

**Methods:**

In this study we evaluated the mutational profile and prevalence of *BRCA* pathogenic/likely pathogenic variants among probands fulfilling the NCCN HBOC testing criteria. We characterized the clinical profile of these individuals and explored the performance of international testing criteria.

**Results:**

A pathogenic/likely pathogenic variant was detected in 19.1% of 418 probands, including seven novel frameshift variants. Variants of uncertain significance were found in 5.7% of individuals. We evaluated 50 testing criteria and mutation probability algorithms. There was a significant odds-ratio (OR) for mutation prediction (p ≤ 0.05) for 25 criteria; 14 of these had p ≤ 0.001. Using a cutoff point of four criteria, the sensitivity is 83.8%, and the specificity is 53.5% for being a carrier. The prevalence of pathogenic/likely pathogenic variants for each criterion ranged from 22.1% to 55.6%, and criteria with the highest ORs were those related to triple-negative breast cancer or ovarian cancer.

**Conclusions:**

This is the largest study of comprehensive *BRCA* testing among Brazilians to date, and the first to analyze clinical criteria for genetic testing. Several criteria that are not included in the NCCN achieved a higher predictive value. Identification of the most informative criteria for each population will assist in the development of a rational approach to genetic testing, and will enable the prioritization of high-risk individuals as a first step towards offering testing in low-income countries.

## Introduction

Breast cancer is the most prevalent cancer among women, with about 5–10% of all cases caused by inherited germline pathogenic variants in cancer predisposition genes [1]. *BRCA1* and *BRCA2* (collectively named *BRCA* hereafter) are the main genes causing hereditary breast and ovarian cancer syndrome (HBOC), and are also associated with an increased risk of prostate and pancreatic cancers [2]. Pathogenic variants in the *BRCA* genes are the most powerful predictors of developing breast and ovarian cancer, with a 40–80% lifetime risk of developing breast cancer, and 11–50% of developing ovarian cancer, respectively [3]. HBOC patients may benefit from risk-reducing surgery (mastectomy and salpingo-oophorectomy), chemoprevention and enhanced surveillance approaches [4], therefore identification of carriers is crucial for cancer prevention and control. Thus, genetic cancer risk assessment (GCRA) and genetic testing should be an option for patients whose personal and/or family history is suggestive of HBOC syndrome [5].

Brazilian individuals with suspected HBOC syndrome have limited access to GCRA and genetic testing, which has only become available recently for patients with private health insurance. The majority (about 70%) of the population relies on the public health care system, wherein genetic counseling is only available in a few reference centers and genetic testing is not offered [6]. In addition, only a few studies reported on comprehensive *BRCA* variant screening [7–10]. Finally, while international criteria (i.e. NCCN-based), have been routinely used to identify candidates for genetic testing in Brazil, there has been no assessment of the performance of these criteria in this specific population.

Although there are similarities among *BRCA* testing criteria worldwide, there is no consensus and it is reasonable to hypothesize that the prevalence of pathogenic variants will differ according to the population being studied and the testing criteria used [11]. The purpose of this study was to evaluate the prevalence of *BRCA1* and *BRCA2* pathogenic/likely pathogenic variants and clinical profiles of individuals fulfilling the NCCN HBOC testing criteria in Southern Brazil with the aim of assessing the performance of these and other widely used international testing criteria in this specific population.

## Materials and methods

### Subjects and ethical aspects

Study subjects were recruited in the city of Porto Alegre, Southern Brazil, from cancer genetics clinics in Hospital de Clínicas de Porto Alegre (a public general hospital) and private health care offices. All participants were unrelated and had to fulfill *BRCA* testing criteria according to the National Comprehensive Cancer Network (NCCN) guidelines (version 2.2014) for inclusion in the study. The fulfillment of NCCN HBOC criteria was assessed through independent review of all pedigrees by at least two of the authors. This project was approved by the Institutional Review Board from the Hospital de Clínicas de Porto Alegre and all individuals provided written or verbal consent for *BRCA* testing and all participants received pre- and post-test genetic counseling. Since our sequencing data contain sensitive patient information, and patients did not consent to full disclosure of all sequencing information, we have some ethical limitations regarding the raw data access. Specific data requests can, however, be analyzed in a case-by-case basis, and might be available upon request.

### Clinical data and pedigrees

The family history of each participant was recorded, including first-, second- and third-degree relatives on both the maternal and paternal sides of the family, and spanning at least three generations. Confirmation of the personal and family history of cancer was attempted in all cases, through pathology and medical reports as well as death certificates. Clinical data (gender, birthplace, age at cancer diagnosis, tumor type, immunohistochemistry and histology data) were obtained from a review of medical records. For cases in which the age at diagnosis was unavailable, it was conservatively estimated to be older than 60 years. Both lineages were assessed and all pedigrees were evaluated in a single lineage (maternal or paternal), unless there was explicit information on patient adoption. Otherwise, unless specified by the guideline, pedigrees were restricted to three generations. Fallopian tube and primary peritoneal cancers were included as ovarian cancers, and both invasive and *in situ* breast carcinomas were included.

Geographic distribution of proband’s birthplaces were analyzed in R using the packages *maptools* and *maps*, version 3.2.3 (https://www.R-project.org/) [12], along with geographic coordinates obtained from Google Maps (google.com.br/maps).

### Genetic testing

Whenever possible, the initial testing was carried out in a family member with breast or ovarian cancer (affected individuals). However, in some families, only an unaffected individual (or an individual with a diagnosis other than breast and/or ovarian cancer) was available for testing. Sequencing analysis of the entire coding region of the *BRCA1* and *BRCA2* genes and intron-exon junctions was performed in all cases, either using next generation sequencing (NGS) or Sanger sequencing. Probands tested at Hospital de Clínicas de Porto Alegre (HCPA) were sequenced either by Sanger dye terminator sequencing or NGS on the Ion Torrent Personal Genome Machine (PGM) platform. Sequencing on the PGM was carried out according to the manufacturer’s instructions using the Ion AmpliSeq *BRCA1* and *BRCA2* Community Panel and Ion AmpliSeq Library kit 2.0. All VUS or likely pathogenic/pathogenic variants identified by NGS in these patients were confirmed by Sanger sequencing. Large genomic rearrangement testing (LGR) of both *BRCA1* and *BRCA2* was done in the majority of probands. All probands tested at HCPA, and those tested in commercial laboratories with Sanger sequencing were evaluated by Multiplex Ligation-Dependent probe Amplification (MLPA), carried out using MRC-Holland commercial kits for *BRCA1* (SALSA MLPA P002-D1) and *BRCA2* (SALSA MLPA P045-B3) according to the manufacturer’s instructions. Multiplex PCR amplified products were separated by capillary gel electrophoresis in an ABI 3500 Genetic Analyzer. Information on copy number was analyzed with Coffalyser Software (MRC-Holland, http://www.mrc-holland.com/). Identified rearrangements were confirmed in an additional independent experiment, performed with confirmatory kits using different probes: SALSA MLPA P087 for *BRCA1* and SALSA MLPA P077 for *BRCA2*. Probands recruited from private genetic counseling clinics which had genetic testing done by NGS were all tested also by MLPA if the NGS platform did not allow LGR detection. In cases tested with a NGS workflow that allowed the detection of LGR, all identified LGR were confirmed by MLPA. *BRCA* sequencing results (single nucleotide variants and small insertions and deletions only, not including any clinical data or clinical comparisons) of 193 cases were previous published [13].

### *In silico* analyses

In order to estimate the impact of variants of uncertain significance on protein structure, function and evolutionary conservation, we used three different predictors: PredictSNP [14], which combines the results of six prediction tools (MAPP, PhD-SNP, Poly-Phen1, Poly-Phen2, SIFT), AlignGVGD, [15] and MutationTaster [16].

Variants were named following Human Genome Variation Society (HGVS) nomenclature guidelines. The biological significance of all variants were assessed using the databases: CLINVAR (https://www.ncbi.nlm.nih.gov/clinvar), BRCA Share (formerly known as UMD; http://www.umd.be), LOVD (http://databases.lovd.nl/shared/genes), ARUP (http://arup.utah.edu/database/BRCA) and BRCA Exchange (http://brcaexchange.org). Novel variants were classified according to the ACMG [17] guidelines. The population databases 1000 Genomes [18] (http://www.internationalgenome.org/), ExAC [19] (http://exac.broadinstitute.org/), FLOSSIES (https://whi.color.com) and AmbryShare (https://share.ambrygen.com) were consulted to evaluate the population frequency of variants of uncertain significance with full knowledge that the Brazilian population is vastly underrepresented in these databases. All likely pathogenic variants were considered with pathogenic variants (and collectively named “pathogenic variants” hereafter) for analysis of selection criteria, as is standard practice in GCRA.

### Testing criteria

Pedigrees were evaluated for the fulfilment of several different *BRCA* testing criteria, including National Comprehensive Cancer Network (NCCN)[20] criteria, the American Society of Clinical Oncology (ASCO) [21, 22], American Society of Breast Surgeons (ASBS) [23], Society of Gynecologic Oncology (SGO) [24], Spanish Society of Medical Oncology (SEOM) [25], The Institute of Cancer Research (ICR; NHS Foundation Trust) [26] and the Brazilian National Supplementary Health Insurance Agency (ANS) [27], which uses NCCN-based testing criteria and provides access to genetic testing for patients with private health insurance only. As some of the guidelines have overlapping criteria, in total we analyzed 50 distinct criteria. We used additional tools to assess predicted pathogenic variant prevalence or empiric prior probabilities of carriage of pathogenic variants, such as the Manchester [28] and PennII [29] models and the Myriad Mutation Prevalence Tables [30]. To dichotomize the values provided by these models, we set 10% as a minimum probability of being a carrier as a determinant criteria to offer genetic testing, based on the widely accepted ASCO guidelines [21]. All testing criteria, references and specifications are summarized in S1 Table.

### Statistical analysis

Fisher’s exact test was used to determine if there was a significant association between the presence of a pathogenic variant and clinical and pathologic features. Logistic regression was used to determine the odds ratios (ORs) and 95% confidence intervals (CIs). The OR value was used to evaluate the association of each criteria with carrier status.

Receiver operating characteristic (ROC) curves and area under the curve (AUC) was used to determine how many criteria should be fulfilled to predict mutation, and we used 3 different criteria sets: 1) considering all evaluated criteria; 2) only criteria with p ≤ 0.05 on OR analysis; and 3) only criteria with p ≤ 0.001 on the same analysis. The cut point was chosen to reach maximum sensitivity and specificity, considering the value of sensitivity > 70%.

The Mann–Whitney test was used to evaluate the difference between ages at diagnosis. All statistical tests were 2-sided. All analyses were performed using Statistical Product and Service Solutions (SPSS) software version 18.0 (IBM).

## Results

### Clinical features of the cohort

**Fig 1:**
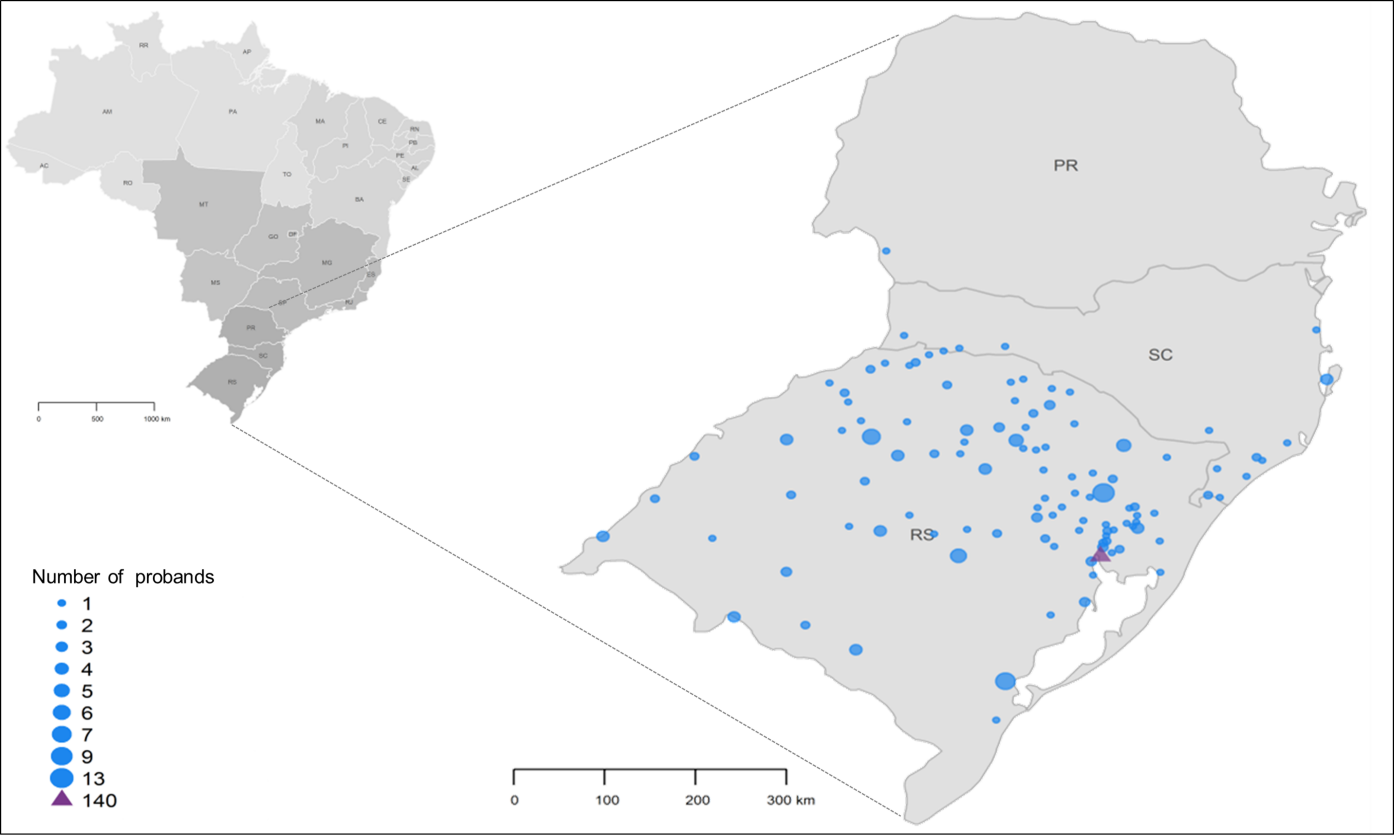
Geographic distribution of probands included in this study in the southern region of Brazil. The size of the dots corresponds to the number of probands from each location. The purple triangle represents the State’s capital, Porto Alegre, from which 140 probands derive. RS, Rio Grande do Sul; SC, Santa Catarina; PR, Paraná.

A total of 418 unrelated probands were enrolled in this study. All participants were recruited in Porto Alegre, the southernmost capital of Brazil. As depicted in Fig 1, 94.6% of individuals were born in the Southern region of Brazil. Eight probands had non-Brazilian nationalities including: Uruguay (3), the Republic of Armenia, Colombia, China, Russia and Romania (1 each). Most individuals were breast cancer (BC) affected women (N = 330, 79%), while only 37 (8.8%) had been diagnosed with ovarian cancer (OC). Fifty-four (13%) individuals were not affected by cancer, but fulfilled NCCN testing criteria. The mean age at diagnosis was 41.6 (standard deviation, SD = 10.5) years for BC and 45.3 (SD = 13.9) years for OC patients. Among patients with bilateral BC (14 synchronous and 27 metachronous primaries), the mean age at diagnosis was 43.1 (SD = 11.4) and 49.0 (SD = 13.3) years for the first and second tumors, respectively. The characterization of tumor types, ethnicity, BC receptor status, BC and OC histology and age at diagnosis are summarized in Table 1.

**Table 1:** Clinical and pathologic features in *BRCA1* and *BRCA2* carriers of pathogenic variants and non-carriers.

			All participants (N = 418)		Carriers of pathogenic variants (N = 80)		Non-carriers* (N = 338)		P-value
			N	%	N	%	N	%	Carriers *vs* Non-carriers
**Gender**									
	Female		408	97.6	78	97.5	330	97.6	1.00
	Male		10	2.4	2	2.5	8	2.4	
**Birthplace**^a^									
	Brazil		365	97.9	70	98.6	295	97.7	0.419
		Southern Brazil	353	94.6	66	93.0	287	95.0	
		Others	12	3.2	4	5.6	8	2.6	
	Other countries		8	2.1	1	1.4	7	2.	
**Cancer affected**									
	Breast cancer only		319	76.3	59	73.8	260	76.9	0.030
		Single tumor	255	61.0	45	56.3	210	62.1	
		Bilateral	41	9.8	11	13.8	30	8.9	
		Ipsilateral	17	4.1	2	2.5	15	4.4	
		Male	6	1.4	1	1.2	5	1.5	
	Ovarian cancer only		26	6.2	8	10.0	18	5.3	
	Breast and ovarian cancer		11	2.6	6	7.5	5	1.5	
	Other^b^		8	1.9	0	0.0	8	2.4	
**Cancer unnaffected**			54	13.0	7	8.7	47	13.9	
**Ashkenazi Jewish ethnicity**									
	Yes		13	3.1	5	6.3	8	2.4	0.082
	No		405	96.9	75	93.7	330	97.6	
**Breast cancer receptor status**^c^									
	TNBC		78	29.8	34	59.7	44	21.5	<0.0001
	HR-positive/HER2-negative		139	53.0	20	35.0	119	58.0	
	HR-negative/HER2-positive		17	6.5	3	5.3	14	6.8	
	HR-positive/HER2-positive		28	10.7	0	0	28	13.7	
**Histology of breast cancer**^d^									
	IDC		210	72.4	48	87.3	162	68.9	0.047
	DCIS		19	6.6	0	0.0	19	8.0	
	ILC		19	6.6	1	1.8	18	7.7	
	LCIS		3	1.0	0	0.0	3	1.3	
	IDC + ILC		6	2.0	0	0.0	6	2.6	
	Other		33	11.4	6	10.9	27	11.5	
**Histology of ovarian cancer**									
	Serous		10	38.5	6	75.0	4	22.2	0.026
	Non-serous		16	61.5	2	25.0	14	77.8	
**Age at diagnosis (years)**^e^			**Mean (SD)**		**Mean (SD)**		**Mean (SD)**		
	Breast cancer		41.6 (10.5)		39.7 (10.3)		42.1 (10.5)		
		Single tumor	40.8 (9.8)		40.3 (10.2)		40.9 (9.7)		
		Bilateral^f^	43.1 (11.4)		35.3 (8.8)		46.0 (11.0)		
		Ipsilateral^f^	43.6 (12.2)		38.5 (0.7)		44.3 (12.9)		
		Male	60.5 (13.8)		64.0 (0.0)		59.8 (15.3)		
	Ovarian cancer		45.3 (13.9)		47.9 (8.7)		44.2 (15.8)		
	Other		47.4 (9.7)		-		47.4 (9.7)		

TNBC, triple-negative breast cancer; HR, hormonal receptor; IDC, invasive ductal carcinoma; DCIS, ductal carcinoma *in situ*; ILC, invasive lobular carcinoma; LCIS, lobular carcinoma *in situ*.

* Non-carrier group is composed of WT individuals and VUS carriers.

**(a)** Birthplace data was missing for 45 individuals

**(b)** Other tumors are: endometrial cancer (1), colorectal cancer (1), renal clear cell carcinoma (1), thyroid cancer (1), pancreatic cancer (2) and melanoma (2)

**(c)** Receptor status data was not available for 68 breast cancer patients

**(d)** Histology data was not available for 40 patients. In (c) and (d) only the status of the first tumor was considered

**(e)** Patients diagnosed with both breast and ovarian cancer were excluded from this analysis

**(f)** Only the age at the first diagnosis was considered.

### Pathogenic variants identified in *BRCA1* and *BRCA2*

Almost half of all *BRCA* testing was performed in Brazil (48.8%), with the remaining tests performed in the United States of America (50.5%), Canada, Spain, and Switzerland (collectively 0.7%). For *BRCA* sequencing, NGS was the method of choice to sequence 88.3% (369/418) of all patients, and the entire coding region of both genes was covered for all probands (data not shown).

Eighty-three pathogenic variants in the *BRCA1* and/or *BRCA2* genes were identified in 80 of the 418 probands (19.1%). When only cancer affected probands are considered the prevalence of pathogenic variants rises to 20%, while among cancer unaffected probands with a suspicious family history (i.e. a family history characterized by the presence of specific cancer types and ages, leading to the suspicion of hereditary breast and ovarian cancer syndrome, according to HBOC-NCCN criteria), the prevalence was close to 13%. Among breast and ovarian cancer patients the detection rate of pathogenic variants was 19.7% and 37.8%, respectively.

**Fig 2:**
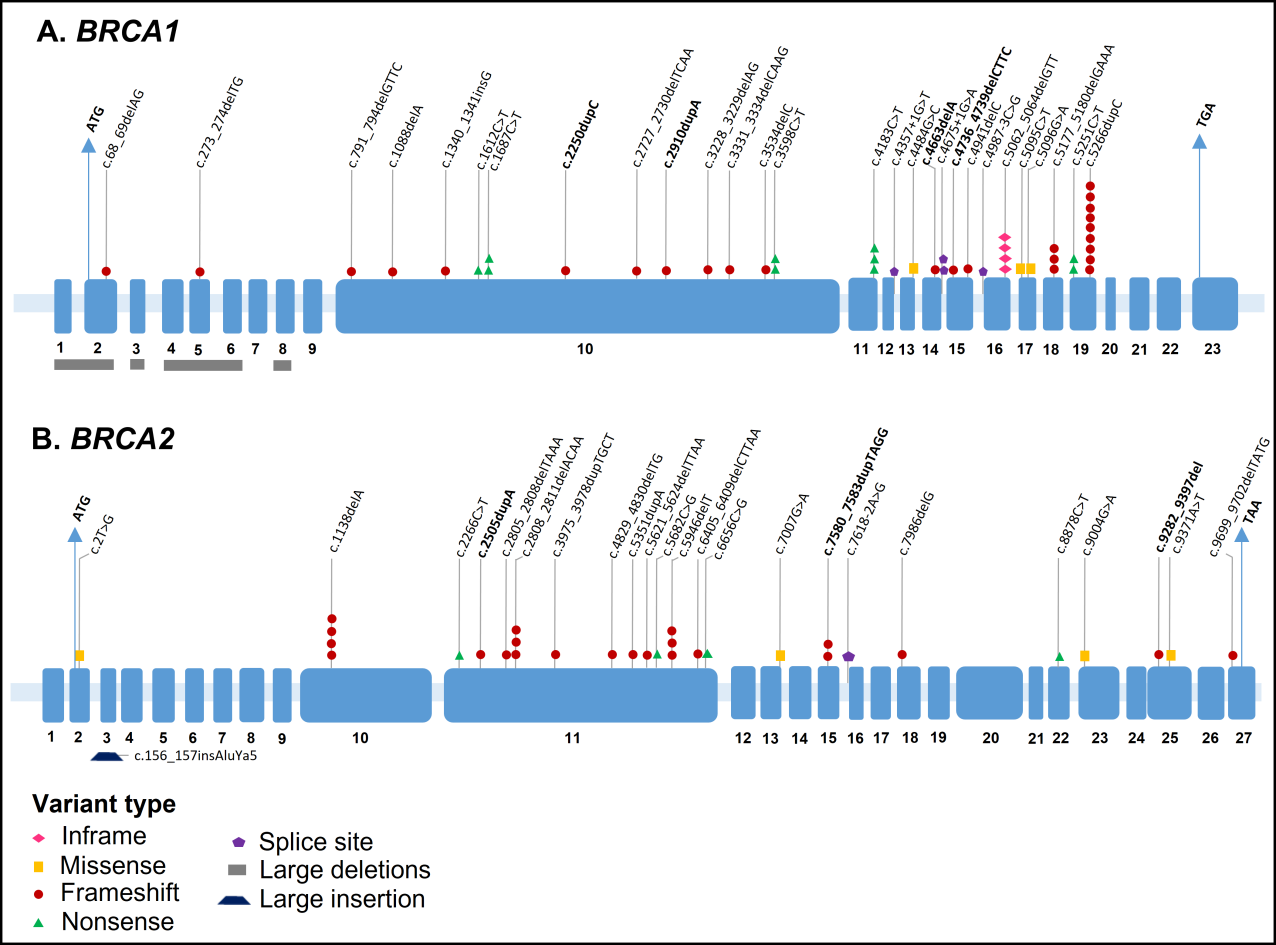
Diagrams of the *BRCA1* and *BRCA2* genes, indicating the position of pathogenic variants identified among all 418 individuals tested. Exons are indicated by blue boxes and numbered according to the Locus Reference Genomic (LRG) description. Different symbols represent the type of variant, and each symbol indicates one germline carrier. Novel variants, described here for the first time are in bold. The ATG sites and termination codons of both genes are indicated by arrows.

Fig 2 depicts the 83 pathogenic variants identified in this cohort, corresponding to 56 unique pathogenic variants carried by 80 probands. In *BRCA1*, four distinct LGR and 28 different pathogenic SNVs and small insertions and deletions (indels) were identified in 51 probands, representing 61.4% of all pathogenic variants found in the study. In *BRCA2*, only one LGR was identified (the Portuguese founder pathogenic variant c.156_157insAluYa5) and 23 distinct pathogenic SNV/indels were found in 31 probands. Of interest, two double heterozygotes (DH), carrying one *BRCA1* and one *BRCA2* pathogenic variant were identified. The first patient was heterozygous for *BRCA1* c.4357+1G>T and *BRCA2* c.6405_6409delCTTAA, while the second patient was heterozygous for a *BRCA1* LGR (deletion of exons 4–6) and *BRCA2* c.9004G>A. In addition, we identified a patient carrying two heterozygous *BRCA2* pathogenic variants (c.8878C>T and c.9699_9702delTATG). All pathogenic variants identified, along with their predicted protein sequences and associated personal history of cancer are described in S2 Table.

Complete *BRCA1* and *BRCA2* LGR analysis was done for 340 (81.3%) probands, and a total of 336 (98.8%) individuals had a wild type result. Forty-eight probands with a pathogenic variant detected by sequencing, and 19 probands with a wild type (WT) sequencing result chose not to proceed with LGR testing. In 11 probands, LGR testing was incomplete, including only *BRCA1* or five specific *BRCA1* rearrangements (3.8 kb deletion in exon 13, 510 kb deletion in exon 22, 7.1 kb deletion in exons 8–9, 26 kb deletion in exons 14–20, and a 6 kb insertion in exon 13).

In eight probands, seven unique novel frameshift variants were identified: c.2250dupC (p.Met751Hisfs), c.2910dupA (p.His971Thrfs), c.4663delA (p.Arg1555Glyfs) and c.4736_4739delCTTC (p.Pro1579Leufs) in *BRCA1*; and c.2505dupA (p.Pro836Thrfs), c.7580_7583dupTAGG (p.Gly2529Argfs, in two probands) and c.9282_9397del (p.Asp3095Argfs) in *BRCA2*. These variants were not previously described in the literature nor are they present in the ClinVar, BRCAShare, ARUP/BRCA, KConFab, LOVD and BRCA Exchange databases. Considering all available evidence and the ACMG[17] guidelines, all novel variants were classified as likely pathogenic.

### Clinical features in carriers of pathogenic variants and non-carriers

We compared clinical features between probands with and without pathogenic variants. As described in Table 1, there were no statistically significant differences between these two groups with regards to gender, birthplace and ethnicity. Regarding tumor type, carriers were more frequently affected by both breast and ovarian cancer than non-carriers (p = 0.03). As expected, triple negative breast cancer was more common in carriers, while triple positive tumors were more common in non-carriers (p < 0.0001). The proportion of different breast cancer subtypes was different between groups, with invasive ductal carcinoma (IDC) more common in carriers when compared to non-carriers. Finally, serous ovarian cancers were also more common among carriers, while most non-carriers had other ovarian cancer histologies (p = 0.026).

### Variants of uncertain significance (VUS) identified

Variants of uncertain significance were found in 24 of the 418 probands included in this study (5.7%), including 5 distinct VUS in *BRCA1*, and 18 in *BRCA2* (Table 2). Most were missense variants (73.9%), followed by intronic (17.4%) and synonymous variants (8.7%). Three *BRCA2* VUS (c.1680T>C, c.6271A>G and c.9502-45G>T) were novel and another three (c.2183A>C, c.7007+53G>A and c.9502-40T>A, also in *BRCA2*) were described only in BRCA Exchange, but without any classification. The latter three VUS were present in at least one population database at very low frequencies (< 0.0002). None of the VUS identified have been described in the FLOSSIES database (composed of ~10,000 women older than age 70 years who have never had cancer), AmbryShare, or ARUP/BRCA databases. None of the VUS were co-occurring with pathogenic variants and although MutationTaster suggested that all intronic and silent variants were likely tolerated, one *BRCA1* (c.5242G>C) and two *BRCA2* (c.9227G>A and c.9227G>T) missense variants were classified as deleterious using all three predictors (AlignGVGD, MutationTaster and PredictSNP).

**Table 2:** Classification of the variants of uncertain significance (VUS) found in our cohort according to different databases and their effects as predicted by *in silico* models.

HGVS name		Molecular consequence	ClinVar	dbSNP	BRCAShare	AlignGVGD	MutationTaster	PredictSNP
***BRCA1***								
	c.1258G>T (p.Asp420Tyr)	Missense variant	Conf. Int.^a^	rs80357488	VUS	C15	Polymorphism	Deleterious
	c.2763G>A (p.Gln921 =)	Silent variant	LB	ND	VUS	NA	Polymorphism	NA
	c.3868A>G (p.Lys1290Glu)	Missense variant	VUS	rs80357254	ND	C0	Disease causing	Neutral
	c.4724C>A (p.Pro1575His)	Missense variant	VUS	rs80357052	VUS	C0	Polymorphism	Deleterious
	c.5242G>C (p.Gly1748Arg)	Missense variant	VUS	rs397507245	ND	**C65**	**Disease causing**	**Deleterious**
***BRCA2***								
	c.67+62T>G	Intronic variant	VUS	rs11571574	ND	NA	Polymorphism	NA
	c.710A>G (p.Asp237Gly)	Missense variant	VUS	rs730881506	ND	C0	Polymorphism	Neutral
	c.1244A>G (p.His415Arg)	Missense variant	VUS	rs80358417	VUS	C0	Polymorphism	Neutral
	c.1680T>C (p.Asn560 =)	Silent variant	ND	ND	ND	NA	Polymorphism	NA
	c.2183A>C (p.Asp728Ala)	Missense variant	ND	ND	ND	C0	Polymorphism	Neutral
	c.3321A>C (p.Gln1107His)	Missense variant	VUS	rs397507306	ND	C15	Disease causing	Deleterious
	c.4477G>C (p.Glu1493Gln)*	Missense variant	VUS	rs398122782	ND	C0	Polymorphism	Deleterious
	c.4627A>G (p.Lys1543Glu)	Missense variant	VUS	rs786204239	ND	C0	Polymorphism	Neutral
	c.6271A>G (p.Ser2091Gly)	Missense variant	ND	ND	ND	C0	Polymorphism	Neutral
	c.6467C>T (p.Ser2156Phe)	Missense variant	VUS	rs765575482	VUS	C0	Polymorphism	Deleterious
	c.6988A>G (p.Ile2330Val)	Missense variant	VUS	rs876661032	ND	C0	Polymorphism	Neutral
	c.7006C>T (p.Arg2336Cys)	Missense variant	Conf. Int.^b^	rs431825347	VUS	C0	Polymorphism	Neutral
	c.7007+53G>A	Intronic variant	ND	rs56014558	ND	NA	Polymorphism	NA
	c.9227G>A (p.Gly3076Glu)	Missense variant	Conf. Int.^c^	rs80359187	ND	**C65**	**Disease causing**	**Deleterious**
	c.9227G>T (p.Gly3076Val)	Missense variant	VUS	rs80359187	ND	**C65**	**Disease causing**	**Deleterious**
	c.10250A>G (p.Tyr3417Cys)	Missense variant	Conf. Int.^d^	rs730881600	ND	C0	Polymorphism	Deleterious
	c.9502-40T>A**	Intronic variant	ND	rs563731281	ND	NA	Polymorphism	NA
	c.9502-45G>T**	Intronic variant	ND	ND	ND	NA	Polymorphism	NA

ND, not described; NA, not applicable; LB, likely benign; Conf. Int, conflicting interpretations of pathogenicity in ClinVar:

**(a)** likely benign (2 submitters); VUS (2 submitters)

**(b)** likely benign (1 submitter); VUS (3 submitters)

**(c)** likely pathogenic (1 submitter); VUS (4 submitters)

**(d)** likely benign (1 submitter); VUS (1 submitter). Variants for which all three *in silico* tools predicted a deleterious effect are shown are in bold.

(*) the variant c.4477G>C was found in two individuals.

(**) both variants were found in the same individual. AlignGVGD classifies each variant from C65 (most likely to interfere with function) to C0 (least likely).

### Performance of different genetic testing criteria

Altogether, we evaluated 50 distinct criteria for genetic testing (all published in international guidelines), in addition to the scores of commonly used pathogenic variant prediction tools (PennII and Manchester models and Myriad Mutation Prevalence tables, using 10% as cutoff point), for a total of 54 criteria (S1 Table). Among these, 25 criteria reached a statistically significant odds-ratio of carrying a pathogenic variant (p ≤ 0.05) and the prevalence of pathogenic variants varied significantly among these criteria, from 22.1% to 55.6%, as depicted in Table 3. Criteria with the highest ORs were those related to triple-negative breast cancer or ovarian cancer. Women with both breast and non-mucinous ovarian cancer have 5.4 times the chance of carrying a pathogenic variant, compared to individuals without this phenotype. Patients with both breast and ovarian primary tumors had a 54.5% chance of carrying a *BRCA* pathogenic variant. It is noteworthy that 17.4% of all carriers with an early onset BC (≤ 45 years) had no family history of breast or ovarian cancer. Among early-onset BC patients without bilateral and/or triple-negative tumor and without a family history of cancer (n = 31), the prevalence of pathogenic variants was 6.5%. Also, two VUS carriers belong to this group.

**Table 3:** Performance of testing criteria with significant odds ratios.

Criteria #	Testing criteria	Reference	Prevalence of pathogenic variants(%)*	OR	95% CI	P
**52**	**Familial PennII Score ≥10**	**18**	**22.1**	**7.643**	**1.822–32.062**	**<0.001**
**39**	Family with sister pair with BC and OC, both diagnosed < 50y	9	55.6	5.567	1.460–21.226	0.015
**53**	**Myriad Table Score ≥ 10**	**19**	**35.9**	**5.553**	**3.262–9.455**	**<0.001**
**44**	**Family with 2 BC and/or OC, and at least 1 OC**	**14**	**49.1**	**5.546**	**3.010–10.219**	**<0.001**
**11**	Personal history of BC and non-mucinous OC	15	54.5	5.400	1.605–18.168	0.008
**35**	**Family with ≥2 BC and ≥1 OC at any age**	**9**	**48.9**	**5.195**	**2.717–9.932**	**<0.001**
**10**	**Personal history of triple negative BC diagnosed ≤60 y**	**10, 11, 12, 13, 16**	**44.2**	**5.014**	**2.871–8.756**	**<0.001**
**9**	**Personal history of triple negative BC**	**15**	**43.6**	**4.881**	**2.8–8.509**	**<0.001**
**23**	**Personal history of BC at any age and ≥1 close relative with OC**	**11, 13, 16**	**46.2**	**4.382**	**2.207–8.701**	**<0.001**
13	Personal history of non-mucinous OC	15	46.7	4.229	1.482–12.067	0.01
**54**	**Manchester Score ≥ 10**	**17**	**22.5**	**4.014**	**1.685–9.563**	**<0.001**
**36**	**Family with ≥3 BC diagnosed < 50 y**	**9**	**40.0**	**3.595**	**1.993–6.486**	**<0.001**
**50**	**Personal history of BC and relatives with cancer and Manchester Score ≥15**	**15**	**31.1**	**3.502**	**2.105–5.824**	**<0.001**
**51**	Individual PennII Score ≥ 10	18	22.6	3.345	1.479–7.564	0.002
**40**	**Family history of OC (non-mucinous)**	**12**	**38.3**	**3.282**	**1.815–5.936**	**<0.001**
**42**	**Family with ≥3 BC and/or OC**	**14**	**28.4**	**2.935**	**1.764–4.881**	**<0.001**
**12**	Personal history of epithelial OC	10, 11, 12, 13, 16	37.8	2.905	1.421–5.941	0.007
**46**	Family with 2 BC: one bilateral and the other diagnosed < 50 y	14	37.1	2.787	1.337–5.810	0.011
**37**	Family with sister pair with BC, both diagnosed < 50y	9	34.5	2.613	1.405–4.858	0.003
**6**	Personal history of bilateral BC, both diagnosed < 60 y	15	35.5	2.535	1.161–5.532	0.029
**45**	**Family with 2 BC diagnosed < 50 y**	**14**	**28.7**	**2.509**	**1.528–4.119**	**<0.001**
**21**	**Personal history of BC at any age and ≥1 close relative with BC diagnosed ≤50 y**	**11, 13, 16**	**27.4**	**2.395**	**1.459–3.933**	**0.001**
**19**	Personal history of BC and a relative with BC, both diagnosed < 50y	15	29.9	2.258	1.332–3.827	0.003
**22**	Personal history of BC at any age and ≥2 close relative with BC at any age	11, 13, 16	27.3	2.123	1.292–3.489	0.004
**8**	Personal history of BC diagnosed ≤50 y and a limited family history^a^	11, 13, 16	28.0	1.908	1.090–3.340	0.028

The fourteen most significant criteria are in bold (P ≤ 0.001). BC, breast cancer; y, years; OC, ovarian cancer

*Percentage of individuals fulfilling the criteria and carrying a pathogenic variant.

**(a)** “Unknown” or “limited family history” applies to individuals with a unknown history or a family with fewer than two first- or second-degree female relatives living beyond age 45.

ROC curve analyses performed with all 54 criteria demonstrated that the presence of ten or more distinct criteria had 76.3% of sensitivity and 58.6% specificity (AUC 0.739; 95%CI 0.479–0.799) for harboring a pathogenic variant. Considering this cutoff, we found that an individual above it has 4.8 times (95%CI 2.64–8.79) the chance of carrying a pathogenic *BRCA* variant, compared to an individual who fulfills less than 10 of the criteria included here. Indeed, among all individuals fulfilling ≥ 10 criteria, 28.9% carried a pathogenic variant, while those individuals who fulfilled < 10 criteria had only a 7.8% chance of carrying a pathogenic variant.

We repeated this analysis considering only criteria with a statistically significant (p ≤ 0.05) OR, and found that at a cutoff point of six or more criteria, the sensitivity and the specificity for being a carrier of a pathogenic variant is 75% and 60.7%, respectively (AUC 0.773; 95%CI 0.717–0.830).

**Fig 3:**
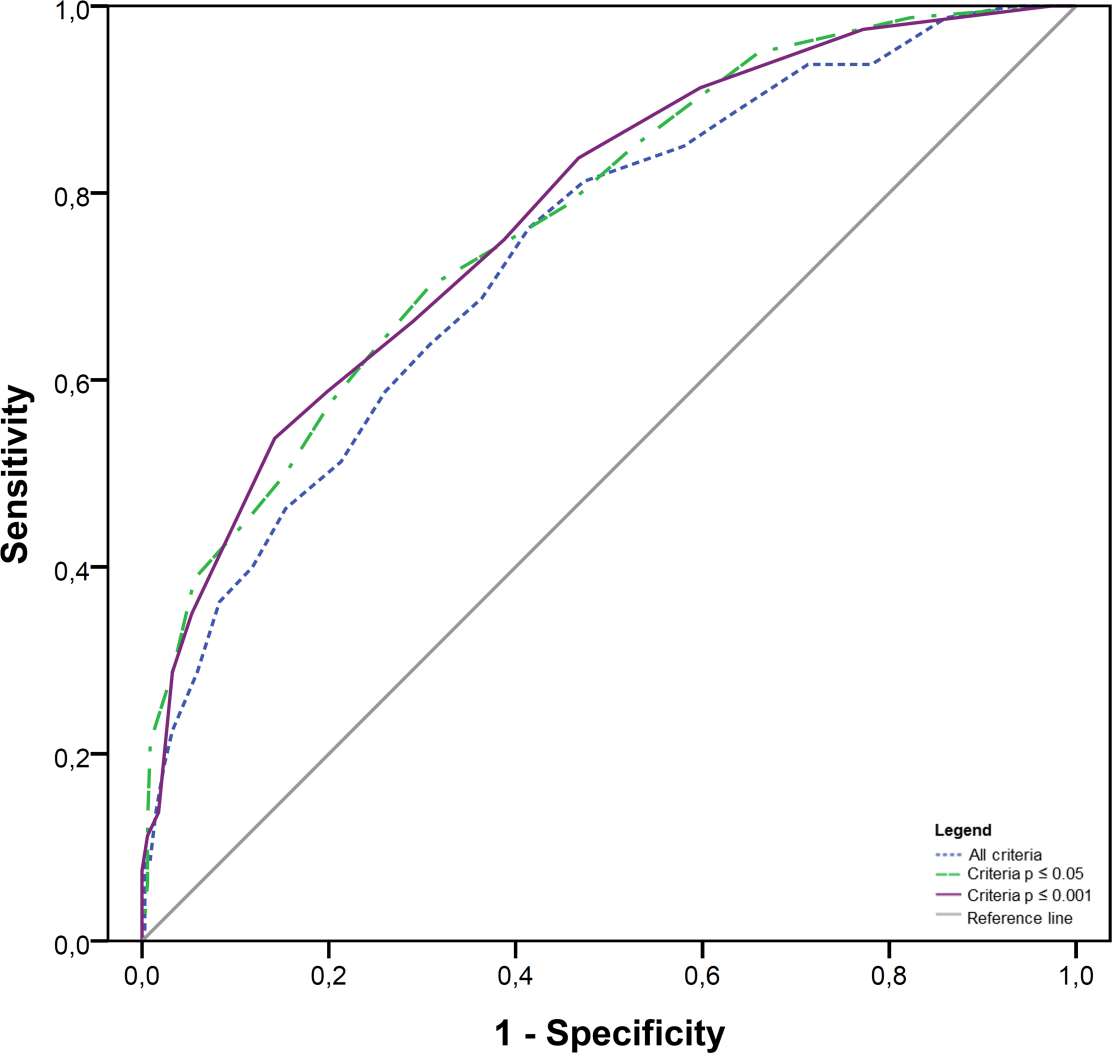
Performance of three distinct criteria sets. Considering all criteria (N = 54, dashed blue line); considering only criteria with p ≤ 0.05 in the OR analysis (N = 25, dashed green line); and considering only criteria with p ≤ 0.001 in the same analysis (N = 14, solid purple line).

Fourteen clinical criteria had a highly statistical significant odds-ratio (p ≤ 0.001) for being a carrier of a pathogenic variant. All carriers of pathogenic variants identified fulfilled at least one of those criteria. In addition, the prevalence of pathogenic variants was directly proportional to the number of these 14 criteria that were fulfilled (S1 Fig). Considering only these 14 criteria, ROC curve analysis showed that at a cutoff point of four or more criteria, the sensitivity is 83.8%, and the specificity is 53.5% (AUC 0.776; p ≤ 0.001) for being a carrier of a pathogenic variant. The chance of carrying a pathogenic variant among probands fulfilling ≥ 4 of the 14 criteria is 5.8 times the chance of an individual with less than four of these criteria (p ≤ 0.001, 95%CI 3.12–11.03). The ROC curves of all three criteria sets are depicted in Fig 3.

It is well known that in a context of low resources there may be a lack of clinically relevant data, as hormone receptor and HER2 status, as well as a detailed family history. Considering this scenario, a ROC curve was used to evaluate the sensitivity of a set of criteria relying only on proband diagnosis, age of onset and limited family history. Using a total of 11 distinct criteria, we show that these criteria were not good predictors of being a carrier of pathogenic variant (AUC 0.580; 95%CI 0.509–0.652, data not shown). Indeed, at a cutoff point of two or more criteria, the sensitivity and specificity of being a carrier of a pathogenic variant are 66.3% 41.4%, respectively.

## Discussion

The identification of a carrier of a pathogenic variant in *BRCA1* or *BRCA2* can significantly impact their medical management, as well as that of at-risk family members, since several cancer risk-reducing strategies are available. In addition, true negative results from cascade testing for relatives of a known carrier provides reassurance and allows application of general population screening guidelines, thereby avoiding unnecessary and costly screening tests and reducing anxiety related to cancer risk. The majority of at-risk individuals in Brazil (the ~70% of the population that relies on the public health care system) do not have access to genetic testing. Thus, national as well as regional molecular profiles of families with the HBOC phenotype have remained largely unknown. Although studies involving Brazilian subjects have been published, most did not include sequencing of the entire coding region and rearrangement testing of both *BRCA1* and *BRCA2* [13, 31]. In addition, many are focused on a specific population and/or involve small cohorts [7–10]. To the best of our knowledge, this is the largest Brazilian study with comprehensive *BRCA* testing to date. It is also the first to evaluate the performance of international testing criteria in this Southern Brazilian population.

In agreement with other studies [32], our data suggests that age at diagnosis (especially for breast cancer), bilateral and/or triple-negative disease, and a diagnosis of ovarian cancer are the findings with the highest predictive value for a testing result positive for a pathogenic variant. Interestingly, in our cohort, epithelial ovarian cancer was diagnosed at a later age in carriers when compared to non-carriers (not statistically significant). A recent study from Azzollini et al [11] highlighted the impact of ovarian cancer cases on the detection rates of pathogenic variants, reporting a mutational prevalence up to 56% among breast and ovarian cancer families. Moreover, breast and ovarian or early-onset ovarian cancer probands with negative family history showed remarkably high detection rates, 43.3% and 26.7%, respectively. Taken together, these findings underscore the importance of testing ovarian cancer patients of all ages, regardless the family history.

The prevalence of pathogenic variants in our cohort was similar to that described in other studies, both in Brazil and in other countries, especially when considering only cancer-affected individuals (prevalence of 20%) [7–10] (Table 4). In addition, also in accordance with other studies from Brazil [7–10] and many other European countries [33], the *BRCA1* c.5266dupC (formerly known as 5382insC) was the most prevalent pathogenic variant, corresponding to 17.6% of all pathogenic variants in *BRCA1*.

**Table 4:** Prevalence of *BRCA* pathogenic variants in Brazilian studies performing comprehensive analysis of *BRCA1* and *BRCA2* among HBOC cohorts.

Reference	Sample size	Inclusion criteria	*BRCA* mutation prevalence
Carraro et al., 2013	54	BC diagnosed < 35 y*	20.4%
Silva et al., 2014	120	HBOC criteria	22.5%
Fernandes et al., 2016	349	HBOC criteria	21.5%
Maistro et al., 2016	100	Epithelial OC*	19.0%
Alemar et al., 2017 (present study)	418	HBOC criteria	19.1%

*These criteria are also considered “HBOC criteria” according to the NCCN v.2014.2.

The overall expected frequency of VUS among *BRCA* tested individuals worldwide is about 7%, but this frequency may vary depending on the patient’s ethnicity, increasing up to 21% in African-Americans [34]. Not surprisingly, considering the predominance of European ancestry in Southern Brazil, VUS were identified in 5.7% of our patients, similar to the prevalence described in North-Americans of European descent (5–6%) [34].

Surprisingly, our rate of novel pathogenic variants (12.5%) identified was lower than previously described in other Brazilian regions: 25% in the cohort reported by Silva et al [8] and 30% in the study of Carraro et al [7]. This again could be due to the higher proportion of Europeans in the population in Southern Brazil, with lower proportions of African- and Amerindian-ancestry than in other Brazilian regions. The lower rate of novel pathogenic variants could be due to the fact that most of the patients studied today worldwide are of European countries or of European descent. Another noteworthy finding from our study is the high prevalence of double heterozygous mutant individuals. In previous large cohorts of Brazilian patients, none of the patients studied was a carrier of more than one germline pathogenic variant in a *BRCA* gene. Double *BRCA1* and *BRCA2* heterozygotes have been described in only a few studies and have been considered rare in most populations [35]. Therefore it is surprising that in our population 2.5% of all carriers were double heterozygotes, if we include one patient carrying two *BRCA2* pathogenic variants. However, because the patient did not present with a phenotype of Fanconi’s Anemia these variants are likely to be in the same *BRCA2* allele (i.e. in cis). The parents were not available for testing, to confirm this hypothesis.

One of the most important determinants of the yield of genetic testing is the testing criteria used. The fact that our overall prevalence of pathogenic variants was similar to that described in other countries indicates that the NCCN criteria, used for patient selection in our study, are performing well in the identification of carriers of pathogenic variants in this group of patients. However, we showed that several criteria not included in the NCCN or in the Brazilian (ANS) testing guidelines (e.g. ASCO criteria, criteria #35—Family with ≥2 BC and ≥1 OC at any age, #36—Family with ≥3 BC diagnosed < 50 y, #37—Family with sister pair with BC, both diagnosed < 50y and #39—Family with sister pair with BC and OC, both diagnosed < 50y, Table 3) had very high odds ratios for carrying a pathogenic variant. This is not unexpected, since these criteria are far more stringent than the NCCN criteria, and suggests that those criteria could be used as a prioritization approach in this population, in a scenario of limited resources. In addition, we observed that individuals fulfilling multiple criteria are more likely to carry a *BRCA* pathogenic variant. Also, the comparison between the predictive performance of distinct criteria sets (Fig 3) shown that the use of a large set of criteria did not improve the performance. In contrast, the use of a smaller set of criteria (N = 14) with high p-values in the OR analysis (p ≤ 0.001) shown better results (AUC 0.776, 95%CI 0.720–0.833). The identification of highly predictive criteria in a specific population could guide establishment of priorities in genetic testing in a scenario of limited resources. Although this is the largest HBOC cohort published in Brazil it is relatively small and local. For this reason, we do not intend to make a formal recommendation, but instead our data aim to raise awareness and discuss the possibility of change the criteria used to decide who should be tested in a specific context of limited resources.

Several limitations must be considered when analyzing the data presented here. First, the cohort of patients studied, although significant in size is probably not entirely representative of the population of Southern Brazil. Brazil is a country of continental dimensions and formed by a very admixed population with contributions from Europeans, Africans and Native Americans in different proportions according to geographic region [36]. Thus, it is possible that the profile of pathogenic variants will be unique in different Brazilian regions. Also, besides patients prospectively recruited, this study also included retrospective data. Moreover, since we recruited patients from high risk clinics, the performance of selected criteria may not be the same for individuals in the general population. Although all participants fulfilled the same clinical criteria (NCCN version 2014.2), they were recruited from different clinics and a heterogeneity in variant testing strategies was present, with some individuals being tested by Sanger sequencing and others by NGS. In addition, LGR analysis was incomplete, mainly due to the fact that until very recently testing in Brazil followed a stepwise approach with sequencing being done first. The lack of LGR analysis in some individuals, as well as the absence of specific testing for the Portuguese founder rearrangement, *BRCA2* c.156_157insAlu, may have underestimated its frequency and could also have impacted the frequency of double heterozygotes. Our LGR prevalence (1.19%), however, is not significantly different from that reported in previous comprehensive Brazilian studies (0.29% [9], 1.7% [8] and 2% [10]), indicating that an underestimation is not likely. Gleason scores were not available for most prostate cancer cases and not all cancers in the proband’s relatives could be confirmed by pathology and medical reports. Finally, the small number of probands fulfilling some of the criteria could have had an impact on the robustness of the respective ORs.

In conclusion, this is the first comprehensive study on the molecular profile of HBOC probands from Southern Brazil. The prevalence of pathogenic variants is similar to that observed in other Brazilian studies and in other countries, and *BRCA1* and *BRCA2* molecular heterogeneity is also as striking as described in most populations. The identification of a significant proportion of double heterozygotes in our cohort reinforces the importance of comprehensive *BRCA* gene testing. Finally, with this study we have demonstrated that a specific subset of clinical criteria are highly predictive of carrying a pathogenic variant in this population. Taken together with the significantly reduced sequencing costs of next generation sequencing, the strategy of identifying criteria which are highly predictive for presence of a pathogenic variant in a specific population could be used as a first step to prioritize genetic testing in a scenario of limited resources.

## Supporting information

S1 Table Prevalence of pathogenic variants in the cohort according to different testing criteria and performance of each criteria. (XLSX)

S2 Table *BRCA1* and *BRCA2* pathogenic variants identified in our cohort, and personal history of cancer of each carrier. (XLSX)

S1 Fig The prevalence of pathogenic variants according to the number of criteria fulfilled, considering only those 14 clinical criteria that had a highly statistical significant odds-ratio (p ≤ 0.001). (TIFF)
