# Correction: *BRCA1* and *BRCA2* mutational profile and prevalence in hereditary breast and ovarian cancer (HBOC) probands from Southern Brazil: Are international testing criteria appropriate for this specific population?

**DOI:** 10.1371/journal.pone.0197529

**Published:** 2018-05-11

**Authors:** Bárbara Alemar, Cleandra Gregório, Josef Herzog, Camila Matzenbacher Bittar, Cristina Brinckmann Oliveira Netto, Osvaldo Artigalas, Ida Vanessa D. Schwartz, Jordy Coffa, Suzi Alves Camey, Jeffrey Weitzel, Patricia Ashton-Prolla

There is an error in the XML that is causing the fourteenth author’s name, Ida Vanessa D. Schwartz, to be indexed incorrectly. The name should be indexed as Schwartz, IVD.

There is an error in the seventh sentence of the fifth paragraph of the Discussion section. The correct sentence is: Therefore, it is surprising that in our population 2.5% of all carriers were double heterozygotes besides one patient carrying two *BRCA2* pathogenic variants.

In the Author Contribution section, Bárbara Alemar (BA) should be listed as one of the persons who took part in the conceptualization of the paper.
