# Supplementary figures and images for: BRCA1 and BRCA2 mutational profile and prevalence in hereditary breast and ovarian cancer (HBOC) probands from Southern Brazil: Are international testing criteria appropriate for this specific population?
Source: PLoS One. 2017 Nov 21;12(11):e0187630. doi: 10.1371/journal.pone.0187630 (PMC5697861; doi:10.1371/journal.pone.0187630)

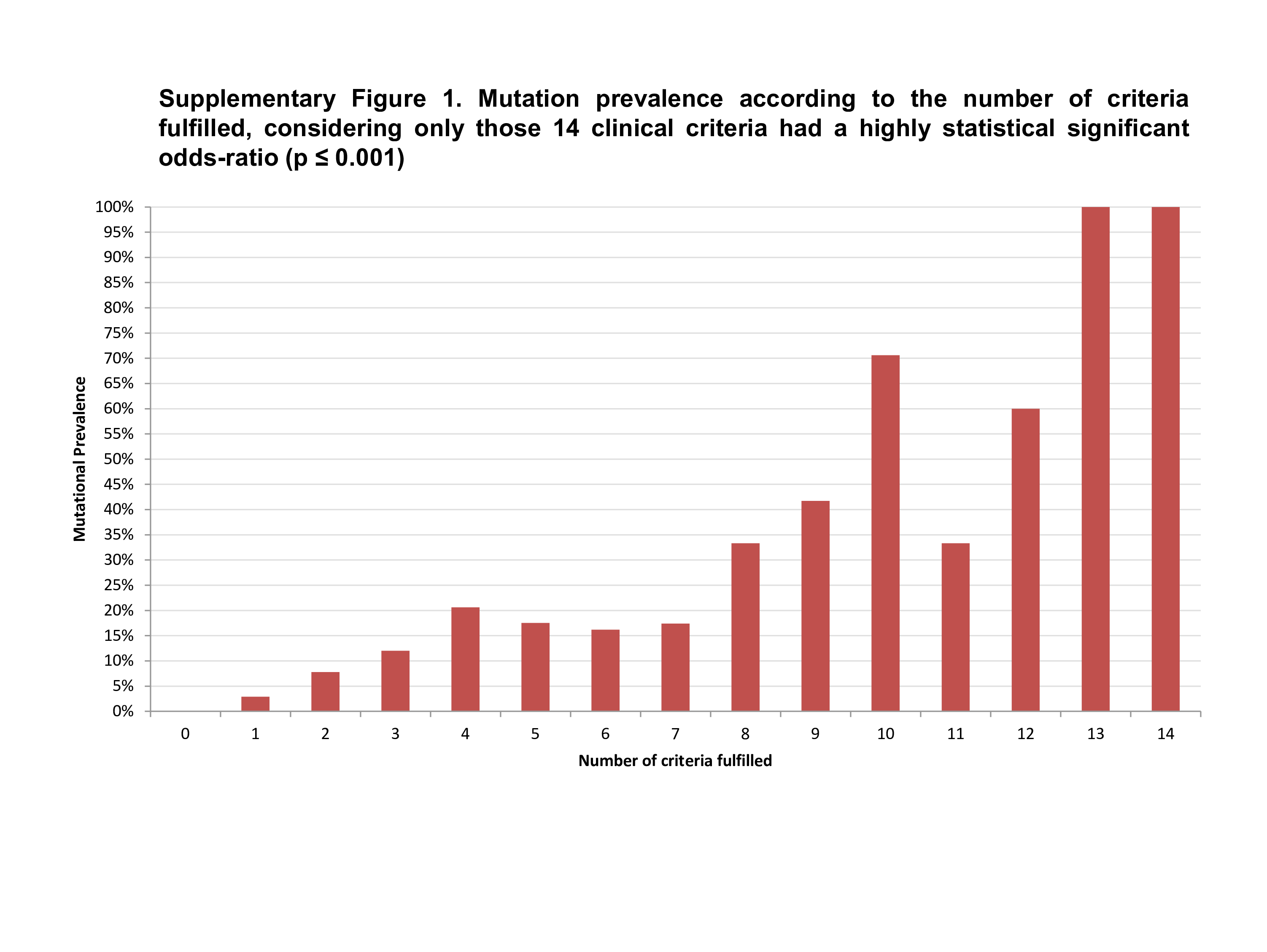

Supplement: S1 Fig — (TIFF) [file pone.0187630.s003.tiff]
